# Collagen organisation in the fibrous joint capsules in the digits of the human hand

**DOI:** 10.1111/joa.70023

**Published:** 2025-07-16

**Authors:** Fiona R. Saunders, Ronald G. Coutts, Richard M. Aspden, Flora Gröning

**Affiliations:** ^1^ Centre for Arthritis and Musculoskeletal Health, School of Medicine, Medical Sciences & Nutrition University of Aberdeen Aberdeen UK; ^2^ Anatomy, School of Medicine, Medical Sciences & Nutrition University of Aberdeen Aberdeen UK; ^3^ MSB Medical School Berlin Berlin Germany

**Keywords:** arthritis, collagen organisation, enthesis, fibrous joint capsule, hand anatomy

## Abstract

Normal function of the hand and, in particular, the finger joints is fundamental to the activities of daily life. Deterioration of hand and finger function can be detrimental and lead to poor quality of life. There are multiple causes of hand and finger dysfunction that can lead to pain and disability. In this review, we will consider the role of collagen and its organization within the finger joint capsules and adjacent entheses, particularly in the proximal interphalangeal joints, and aim to address three questions: (1) What are the main collagen orientations in the interphalangeal joint capsules of the human hand? (2) Is there a relationship between collagen orientation and joint function? (3) How could altering the orientation of collagen fibers affect the functional performance of the joint following injury or surgical intervention? To answer these questions, we will consider the evidence for the main collagen orientations in the finger joint capsules and entheses and investigate the relationships between structure and function. We will then consider how collagen organization is disrupted following injury and what may be potential modulators. This will provide a better understanding of how common surgical interventions affect collagen orientation in the joint capsules and highlight some implications for post‐surgical outcomes.

## Graphical abstract

The function of the finger joints is important for daily life. In this review of the literature, we consider the role of collagen and its organization within the fibrous finger joint capsules of the interphalangeal (IP) joints and investigate the relationships between collagen orientation and joint function.

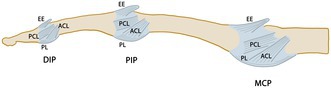

## 1 INTRODUCTION

The maintenance of hand function and, in particular, the delicate functions of the fingers are essential for all activities of daily living and, therefore, any injury to the digits resulting in deterioration of hand function has been demonstrated to have profound consequences on quality of life. Trauma and other orthopaedic disorders of the hand are relatively common and can result in severe pain and disability. In this review, we consider the collagen organization in the capsules of the interphalangeal hinge joints of the digits in the human hand. We explore how changes to the collagen arrangement may affect function since disruption of this arrangement occurs early in the injury cycle, before healing and resolution of the disruption. We will first consider the collagen structure, organization, and function. Then we will describe gross finger joint anatomy and decrease in scale through the joint capsule structure to the microstructure and discuss variations in collagen organization. Evidence for disruption of the collagen architecture, which may exacerbate contracture and cause functional deficits, will be reviewed, and we will discuss some of the potential modulators of the contracture process.

The main questions that will be addressed by this review are:

1. What are the main collagen orientations in the interphalangeal joint capsules of the human hand?
2. Is there a relationship between collagen orientation in the joint capsule and joint function? If so, how are the two related?
3. How do common surgical interventions affect collagen orientation in interphalangeal joint capsules and what are the implications for functional outcomes after surgery?

## 2 COLLAGEN ORGANIZATION AND FUNCTION

Collagen is the most abundant protein in animals. Like a rope, it provides tensile reinforcing to many connective tissues, especially those associated with joints (Aspden, 1994). The triple‐helical collagen molecules are aggregated into fibrils and fibres (Christiansen et al., 2000; Kadler et al., 1996; Kastelic et al., 1978; Orgel et al., 2006) and it is the extended form of the helices and cross‐linking between them that make the fibrils relatively inextensible. The individual collagen type I fibres themselves are very stiff, which helps them resist tensile stress, but their three‐dimensional organisation may add a small amount of additional elasticity (Dutov et al., 2016) for example, in the ligaments (Kirby et al., 1989). In this way, they form rope‐like structures strong in tension but weak in shear and torsion (Hukins & Aspden, 1985), to strengthen tissues as diverse as cartilage and tendon. Every third amino acid within a collagen peptide is a glycine residue, which enables the tight coiling of the structure (Fietzek & Kühn, 1976; Ramachandran & Sasisekharan, 1961; Shoulders & Raines, 2009). Other amino acids commonly found within the collagen structure include proline and hydroxyproline (Ricard‐Blum, 2011). The formation of collagen is complex and will not be discussed in detail here, but a detailed explanation can be found in this excellent paper by Brodsky and Persikov (2005). Another feature of collagen fibre ultrastructure is fibrillar crimps, which cause local deformations and are related to sub‐fibrillar arrangements (Franchi et al., 2008). These crimps are mainly found in large straight fibrils (Franchi et al., 2008). This may account for a slight shortening of collagen fibres in length and make measurements more technically challenging.

There are 28 types of collagen described in vertebrates (Ricard‐Blum, 2011; Shoulders & Raines, 2009), with different forms expressed in different tissues. In Table 1, we show the main forms of collagen that are expressed in musculoskeletal tissues. In ligaments and tendons, collagen types I and III predominate, but in articular cartilage, which is mainly subjected to compressive forces, collagen type II is the main form (Van Der Rest & Garrone, 1991). The other forms of collagen described in Table 1 are less commonly found in tissues and are therefore less well understood. Some forms of collagen are found alongside non‐collagenous proteins and proteoglycans, which form fibrils in a strict hierarchical structure (Shoulders & Raines, 2009). The fibrils have a distinct banding pattern due to the longitudinal molecular packing within the fibrils, which can be observed using high‐power microscopy, such as transmission electron microscopy (Narita et al., 2022; Silver & Trelstad, 1980). Collagen fibrils are elastic, and the ability to deform reversibly (Ricard‐Blum, 2011), but can be modified by glycation, altering their mechanical properties (Avery & Bailey, 2006). Collagen turnover is very slow, with data from atomic bomb carbon pulse experiments showing that there is little turnover in adults (Heinemeier et al., 2016) and collagen fibrils are resistant to most proteases but are degraded by the action of matrix metalloproteinases (MMPs) and collagenases. Collagen fibres are found in highly organised patterns either as fibre bundles or as a network, which is related to the tissue and affects cell‐matrix interactions and the tissue biomechanics (Birk & Bruckner, 2005). The organisation of collagen within a tissue is generally anisotropic and very specific to each tissue. The directions of predominant orientation indicate the directions in which tensile forces are generated within the tissue (Aspden, 1994; Doillon et al., 1985; Hukins & Aspden, 1985; Parry, 1988).

**TABLE 1:** The main forms of collagen expressed in the tissues of the hand.

Structure	Collagen type			
	I	II	III	IV
Dorsal tendon	++++	++	+	+
Palmar tendon	++++	+	+	+
Dorsal capsule	++++	Increases with ageing	+	+
Palmar capsule	++++	−	+	+
Collateral ligaments	++++	Increases with ageing	+	+
Entheses	++++	++++ increases with ageing	+	+
Cartilage	−	++++	−	+
Synovium	+ in sub‐intimal layer	+	+ in pathology	+ in sub‐intimal layer

*Note*: Presence in tissue: ++++ strong presence, +++ medium presence, ++ low presence, + minimal, − not present.

Collagen types I‐III are the main fibre‐forming collagens present in the joint (Aspden et al., 1985; Lewis et al., 1998; Ralphs & Benjamin, 1994). Typically, the fibrils are 30–100 nm in diameter and the fibres, formed from fibrils, are considerably larger than the fibrils (1–20 μm diameter). Fibres tend to be much longer than their diameter, with aspect ratios (length to diameter) of >300–1000:1 (Aspden, 1994; Ushiki, 2002), although collagen fibre lengths are very difficult to measure.

Numerous processes such as repetitive loading, aging, and injury can alter the collagenous structure and organization in different ways. These include altering the ratio of different collagen types within a fiber; renewed expression of collagen types more commonly seen in embryonic development; and changes in collagen distribution and fiber size. Repetitive strain on tissues has been shown to induce significant increases in collagen turnover, sometimes doubling the turnover compared with unstrained tissue (Benjamin et al., 2008; Benjamin & Ralphs, 1996; Duscher et al., 2014; Kjær et al., 2009). Aging has been shown to change the distribution of collagen and affect turnover. In particular, this is demonstrated by the change in the ratio of type I to type III collagens and migration of type II into the tissues, especially where compressive forces are present (Cheng et al., 2011; Ralphs & Benjamin, 1994). Collagen type III is also reported to be more prevalent at sites of injury (Cetti et al., 2003; Eriksen et al., 2002; Maffulli et al., 2000). Glycation causes crosslinking and increased stiffness of collagens with aging, exacerbated by diabetes mellitus (Abate et al., 2010; Ghidella et al., 2002; Ricard‐Blum, 2011). This is particularly evident in regions such as joint capsules, ligaments, and tendons where collagen turnover is low (Abate et al., 2010; Ashour et al., 2018). Disease can also affect the expression of collagen genes with re‐expression of an embryonic form of type II collagen in osteoarthritic articular cartilage (Aigner et al., 1999).

## 3 FINGER JOINT ANATOMY

### 3.1 Gross anatomy of the fingers

**FIGURE 1:**
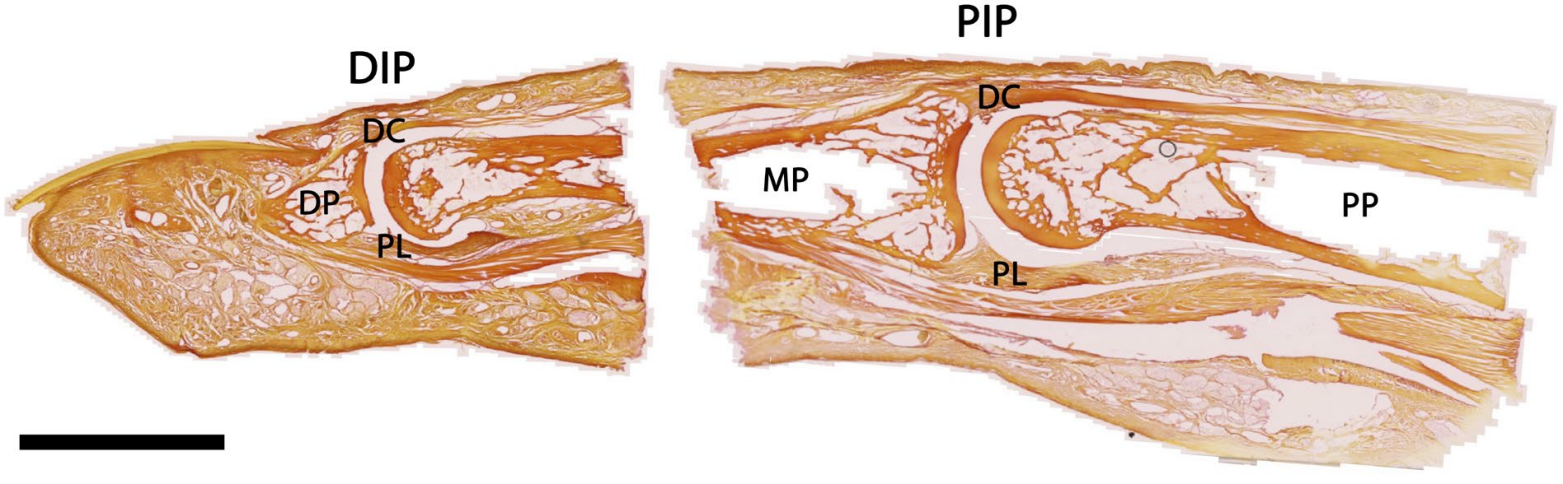
Sagittal sections showing the interphalangeal joints of the fourth digit of a human hand. DC, dorsal capsule; DIP, distal interphalangeal joint; DP, distal phalanx; MP, middle phalanx; PIP, proximal interphalangeal joint; PL, palmar ligament; PP, proximal phalanx. Stained with Van Gieson stain and scanned with a Zeiss Axio Scan Z.1 scanner. Scale bar: 10 mm.

Human fingers are complex structures that allow us to perform very delicate movements. Apart from the thumb, fingers consist of three bones, the proximal, middle, and distal phalanges, which connect to the metacarpals in the hand proper. The phalanges articulate with each other via hinge‐like interphalangeal (IP) joints, with the distal IP joint (DIP) formed between the distal and middle phalanges and the proximal IP joint (PIP) between the middle and proximal phalanges (Figure 1). Each proximal phalanx and metacarpal is articulated via a metacarpophalangeal joint (MCP). These joints are formed between 8 and 10 weeks of embryonic gestation (Gardner & O'Rahilly, 1968; Gray et al., 1957; Mérida‐Velasco et al., 1997; Sadler, 2018). The MCP joint is condyloid in nature and allows for movement within two planes: flexion/extension in the sagittal plane and abduction/adduction in the coronal plane. The fingers can also be circumducted at the MCP joints, meaning that the digits can move in a circular motion, incorporating in sequence flexion, adduction, abduction, and extension. The IP joint, by contrast, is a hinge joint that allows only uniplanar motion (i.e. flexion/extension) in the sagittal plane (Kapandji, 1970). There are a number of ligaments that are involved in the stabilization of the digits and their movements. These will be reviewed in more detail later.

## 4 FINGER JOINT MICRO‐ANATOMY

### 4.1 Joint capsule

The joint capsule is a structure that forms a sleeve around the joint and consists of two layers (Barbe et al., 2009). The outer layer is formed from dense connective tissue comprising interwoven bundles of collagen fibres around the articulating bones and joining to the bones via fibrocartilaginous entheses (Benjamin et al., 1993; Ralphs & Benjamin, 1994). The outer layer is highly innervated but poorly vascularised (Barbe et al., 2009). The inner layer is formed from the synovium and contains synoviocytes, which produce the synovial fluid essential for maintaining lubrication within the joint (Blewis et al., 2007; Ralphs & Benjamin, 1994). The folding of the internal synovial membrane can help with joint lubrication.

Unlike the outer layer, it is poorly innervated but highly vascularized and therefore relatively insensitive to pain (Barbe et al., 2009). The outer and inner layers are held together by a thin but tough subintimal layer acting as a bridging tissue between the superficial subintimal layer and the tendons, ligaments, fat tissue, and the periosteum surrounding the joint (Ricci et al., 2023). Other critical functions of the joint capsule are to provide proprioceptive feedback via sensory nerves, contain the contents of the joint space, and deal with the distribution of mechanical loads through the joints. The most fundamental function of the joint capsule is to hold the joint together and prevent the joint from exceeding the normal range of movement (Ralphs & Benjamin, 1994). The nerves act to protect the joint capsule from injury.

The capsular thickness and collagen fibre orientation vary with location around the joint depending on the stresses experienced (Gacek et al., 2021). The capsule is innervated and accounts for the severe pain that is felt when a digit is subjected to injury (Chen et al., 2000). If the capsule becomes more lax than normal, such as in older age, then the joint is at risk of damage from over‐extension (Ralphs & Benjamin, 1994). It can also contract, however, thus restricting motion of the joint and requiring surgical intervention.

The joint capsules of the MCP, DIP, and PIP joints all have very similar shapes and attachment characteristics. There is a decrease in size of the capsule from the MCP joints to the DIP joints (Buryanov & Kotiuk, 2010; Hutchison & Hutchison, 2010; Karakostis & Lorenzo, 2016). Kuczynski provides detailed descriptions, along with extensive histological imaging of the joint capsule, as far back as 1968 (Kuczynski, 1968) but since then, there has been very little further investigation of the finger joint capsules. Accurate measurements of joint morphology and component structures have proven to be difficult due to the small size and complicated shape of the joints (Fisher et al., 1985).

Ralphs and Benjamin showed that the capsule of the human finger was rich in type II collagen (Benjamin et al., 1993; Ralphs et al., 1991) along with keratan and chondroitin sulphate glycosaminoglycans, more commonly found in articular cartilage. Lewis and coworkers also found consistent staining for collagen types I, III, and IV (Lewis et al., 1998). Abbiati and colleagues reported that the finger joint capsules consist mainly of type I collagen (1995), a finding which was confirmed by Janko et al. (2010). They also described the collagen orientation as highly organised but anisotropic (Janko et al., 2010). This will be discussed in more detail later in this review. Collagen fibres are organised into suprastructures, bundles, and random meshwork that reflect the structure and function of the tissue and of the environment that they are in. The fibre bundles tend to be aligned with the direction of loading, and this leads to anisotropic mechanical properties such as tensile stress and elasticity (Birk & Bruckner, 2005).

### 4.2 Ligaments

**FIGURE 2:**
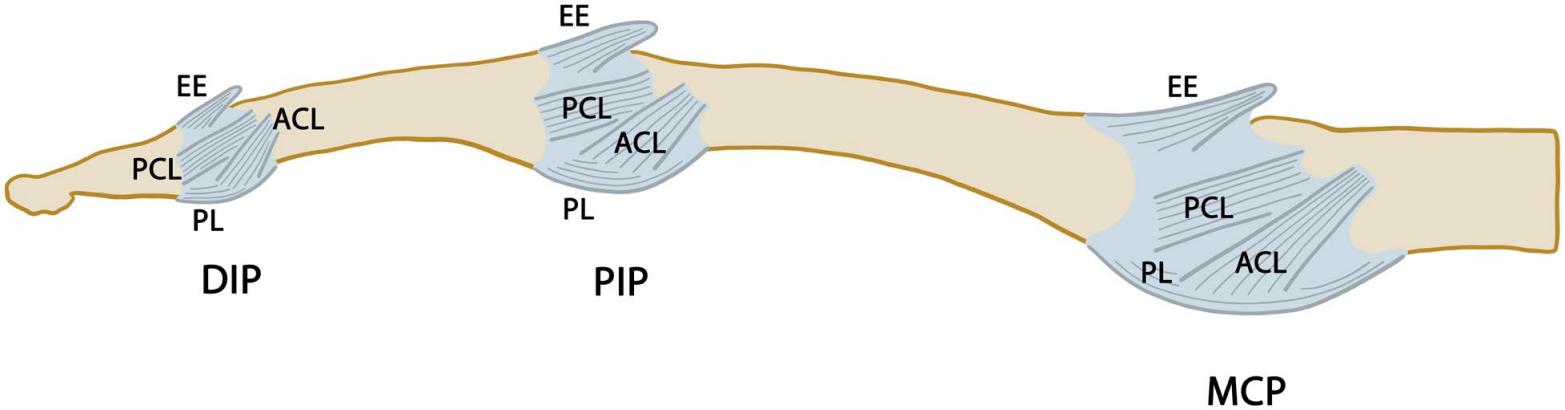
Lateral view of the fibrous finger joint capsules and capsular ligaments. ACL, accessory collateral ligament; DIP, distal interphalangeal joint; EE, extensor expansion; MCP, metacarpophalangeal joint; PCL, proper collateral ligament; PIP, proximal interphalangeal joint; PL, palmar ligament.

**FIGURE 3:**
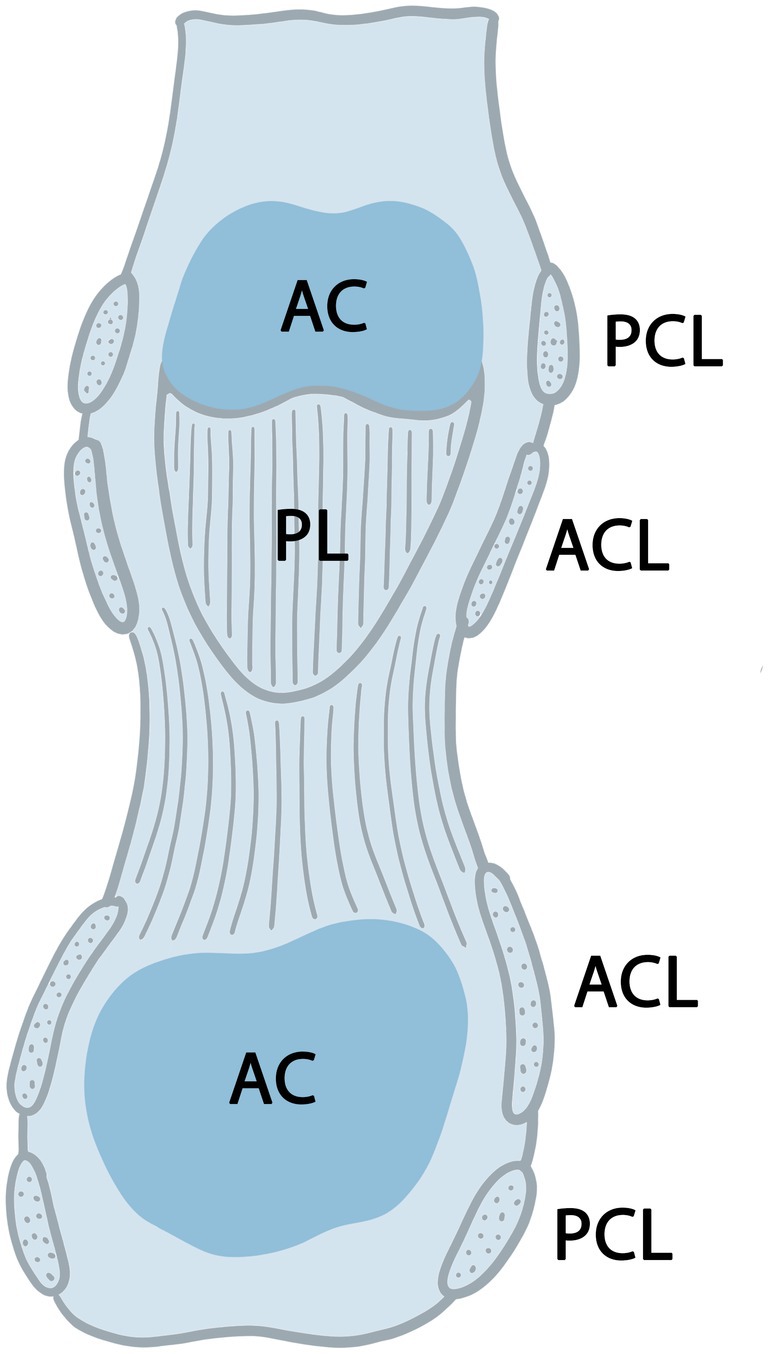
Internal surface of an opened interphalangeal joint capsule. AC, articular cartilage; ACL, accessory collateral ligament; PCL, proper collateral ligament; PL, palmar ligament (adapted from Palastanga et al., 2012).

Ligaments are also critical to the anatomy and function of the fingers, and some are closely related to the fibrous joint capsules. Lewis and colleagues showed that there are several collagen isoforms expressed in the finger joint ligaments, with collagen types I, II, III, and VI being the most commonly found (1998). Each MCP and IP joint has a palmar ligament, two collateral ligaments (sometimes termed proper collateral ligaments) and two accessory collateral ligaments (Figure 2, adapted from (Palastanga et al., 2012)). The main ligament described is the palmar ligament (also referred to in the literature as the palmar plate or volar plate). The palmar ligament's main function is to restrain the extension of the digit (Lewis et al., 1998; Lutter et al., 2023). This ligament attaches to the edge of the articular cartilage at the distal phalangeal base, from where it runs proximally and attaches to the periosteum of the proximal bone, which would be the middle phalanx in the DIP, the proximal phalanx in the PIP, or the metacarpal in the MCP joints respectively (Figure 3) (Watanabe et al., 1994; Williams et al., 1998). Watanabe describes the palmar ligament as having three layers, which have different fibre orientations (1994). The first layer is divided into dense cartilaginous fibres and loose membranous fibres, but these fibres run parallel to the flexor tendon. They describe the second layer fibres as running in the same direction as the flexor tendon, which thickens to the check‐rein ligament, and that in the third layer, the fibre strands are perpendicular to the longitudinal axis of the tendon and bound to fibres of the A3 pulley. Slattery reports that there are transverse, oblique, and longitudinally oriented collagen fibres in the palmar ligament (1990).

**FIGURE 4:**
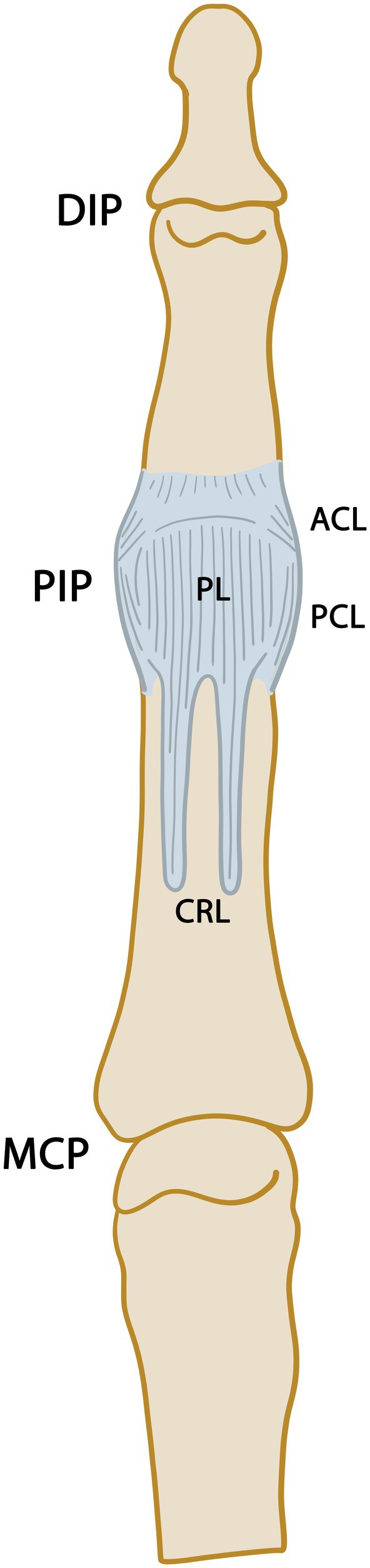
Palmar view of the fibrous joint capsule and capsular ligaments of the proximal interphalangeal joint (PIP). ACL, accessory ligament; CRL, check‐rein ligaments; DIP, distal interphalangeal joint; MCP, metacarpophalangeal joint; PCL, proper collateral ligament; PL, palmar ligament.

The collateral ligaments (also referred to as the proper collateral ligaments in the literature) and the accessory collateral ligaments (Figure 4) have a major role in stabilizing the joint (Kainberger et al., 2018) and span the finger joint lines of both the MCP and PIP joints (Crum et al., 2022). They arise from either side of the IP joints, from the head of the more proximal phalanx and extend distally to the palmar ligament (Rozmaryn, 2017). The fibers are oriented both longitudinally and obliquely (Crum et al., 2022). Due to the orientations of the ligaments, they do not always show up on MRI due to the magic‐angle phenomenon (Kainberger et al., 2018).

**FIGURE 5:**
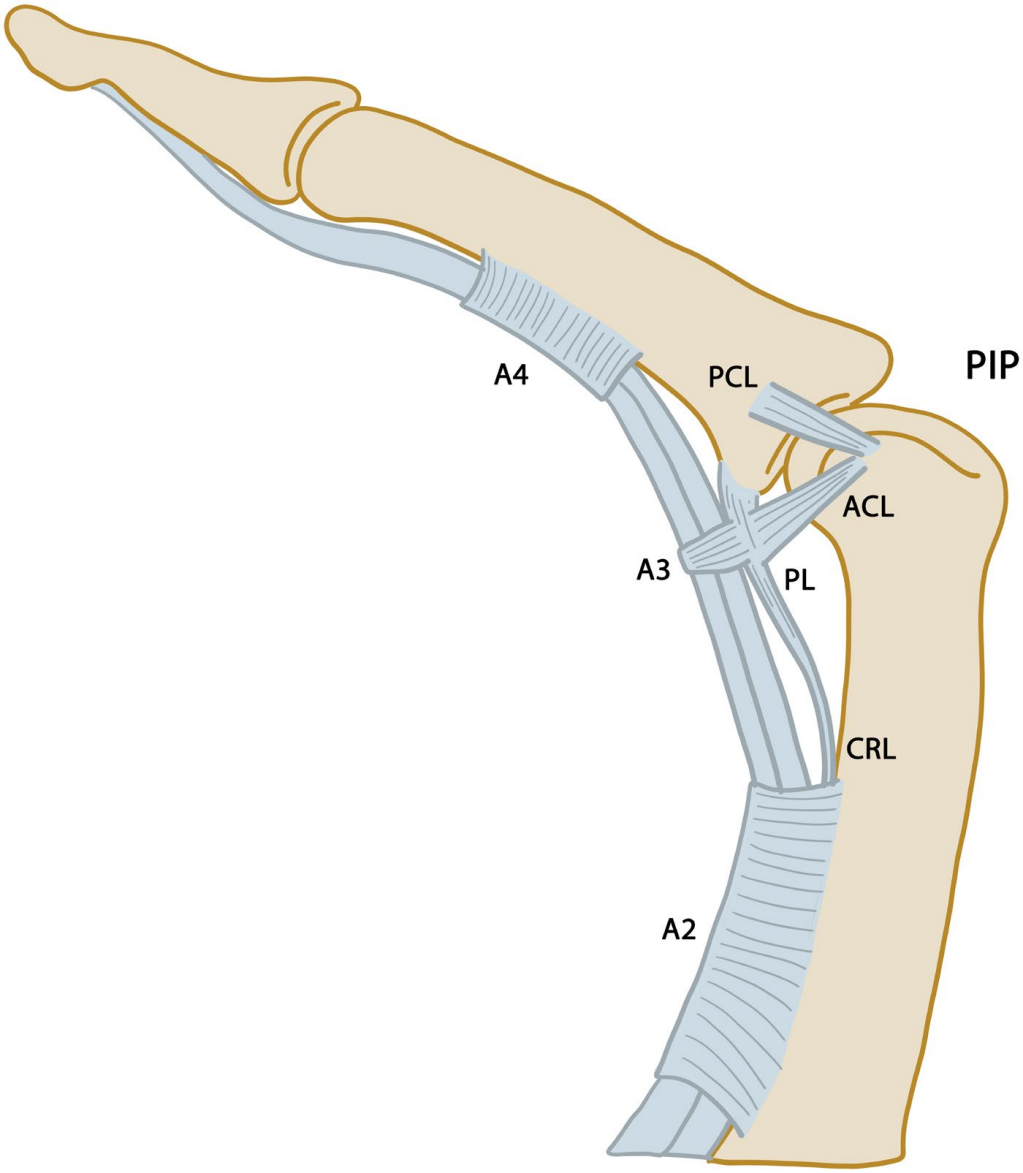
Lateral view of the flexed proximal interphalangeal joint (PIP) showing the relationships between the palmar ligament (PL) and adjacent structures. A2–A4, palmar annular ligaments; ACL, accessory collateral ligament; CRL, check‐rein ligaments; PCL, proper collateral ligament (adapted from Lutter et al., 2023).

There are two extracapsular palmar extensions of the palmar ligament, known as the check‐rein ligaments (Figure 5), which extend proximally on both the medial and lateral aspects of the PIP and attach to the palmar periosteum of the proximal phalanx (Kapandji, 1970; Newton et al., 2019; Slattery et al., 2014; Watanabe et al., 1994). The check‐rein ligaments also protect the vincular blood supply to the palmar anastomosis at the PIP (Bowers et al., 1980; Watson et al., 1979). Additional strength to these ligaments is provided by annular pulleys associated with each digit, namely the C1 and A2 ligaments, in the proximal direction in the PIP joints (Figure 5). If the check‐rein ligaments are restrained, there will be an extension deficit or a flexion contracture will occur (Levaro et al., 2003). The check‐rein ligaments are also involved in the tensioning of the palmar ligament and capsule, allowing the capsule to withdraw and assist in flexion (Bowers et al., 1980). Lewis and colleagues did not find any staining for type II collagen in the palmar ligament (Lewis et al., 1998).

Also, within the MCP joint is a ligament known as the phalangoglenoid ligament. This ligament, along with the check rein ligaments, secures the two sesamoids found in this area to the head of the metacarpal and the base of the proximal phalanx (Benson et al., 2023; Knisely et al., 2023). We have not found evidence that this ligament also exists at the IP joints of the digits, although it is shown for the PIP in the Thieme Atlas of Anatomy (Schünke et al., 2014).

### 4.3 Entheses

Entheses, the sites where tendons, ligaments as well as the fibrous joint capsule attach to bone, are sites where mechanical loads are transmitted from the soft tissues of the capsule or ligaments to the bone (Benjamin et al., 2006; Benjamin & Ralphs, 1998). They can be either fibrocartilaginous in structure or fibrous. Benjamin and Ralphs report that type II collagen may be present (Benjamin & Ralphs, 1998) but others have also documented collagens type I, III, V, VI, IX, X, XI, XIV (Lu & Thomopoulos, 2013; Rossetti et al., 2017). The collagen fibres are highly organised at the entheses, and fibres blend with the mineralized fibres of the bone (Rossetti et al., 2017; Schwartz et al., 2012). The fibrous capsule is attached to the bone via collagen bundles, and these attachments are fibrocartilaginous entheses (Ralphs & Benjamin, 1994). Furthermore, linking the musculoskeletal interface between the capsule to the bone are Sharpey's fibres (Al‐Qtaitat et al., 2010). These fibres are bundles of collagen fibres that serve to connect the periosteum and the bone (Aaron, 2012). These have been widely described in the tooth as fibres within the periodontal ligament (Aaron, 2012). They are also typically found in cranial sutures and sites of muscle to bone attachments (Jones & Boyde, 1974).

## 5 COLLAGEN ORIENTATION IN THE FINGERS

### 5.1 Relevance of capsular collagen orientation

The orientation of collagen within the joint capsule determines the strength, quality, and functionality of the tissue and how well the tissue will repair itself in response to damage (Picu et al., 2018). Collagen fibres are strongest along their dominant orientation, which is determined by the loading applied to the tissue. However, repaired collagen structures (e.g. following injury or surgery) are generally much less organised and can generate contraction across the fibre orientation (Tomasek et al., 2002). Therefore, the orientation of the collagen fibres should be taken into consideration for surgery, as incisions across the fibres are likely to result in poor wound healing (Carmichael, 2014; Ní Annaidh et al., 2012).

We will now discuss in detail each of the principal collagen orientations in the different parts of the fibrous joint capsule in the PIP and DIP joints.

### 5.2 Dorsal capsule

The dorsal capsule collagen orientation predominantly follows the same directions as the overlying tendon of the extensor digitorum, which lies in a proximal to distal orientation. At the macroscopic level, the two structures become indistinguishable (Adi et al., 2017; Ralphs & Benjamin, 1994; Slattery, 1990). On the dorsal surface, the capsule and the extensor expansion emerge from a fibrocartilaginous attachment at the narrow rim of the articular cartilage of the more distal phalangeal base and to the bones (Gad, 1967). The capsule then curves proximally around the synovial sulcus and attaches to the shaft of the proximal phalanx via a fibrous attachment (Kuczynski, 1968). A meniscal‐like structure penetrates into the joint space and, here, the collagen becomes less organized (Fisher et al., 1985). Some papers refer to this structure as a synovial fold (Benjamin et al., 1993; Ralphs & Benjamin, 1994), which would be a more accurate description of this structure. However, none of the papers mentioned above give any angle measurements for the collagen orientation, and there is no further information in the literature.

### 5.3 Palmar capsule

In the palmar ligament, the collagen orientations are different from those observed in the dorsal capsule. The flexor tendons are separated from the capsule by the tendon sheath to enable sliding of the tendons during joint movement. The tendon sheath is an indistinguishable part of the capsule where it meets the palmar annular ligaments (A1–A5) (Gad, 1967). The collagen fibres in these annular ligaments lie in a clear transverse orientation (Figure 5). The crucifix ligaments (C0–C3) lie at 45 degrees to the flexor tendon and there is no clear separation from the capsule (Figure 5). Both the annular and crucifix ligaments prevent bow‐stringing of the flexor tendons by holding them in position during flexion (Kapandji, 1970; Parellada et al., 1996). The capsular collagen orientation at the annular ligaments is initially transverse (Kapandji, 1970) but as we move towards the dorsal surface the orientation changes to a more proximal‐distal direction following the capsule. However, as we move from superficial to deep layers, the dominant orientations become less distinct (Lutter et al., 2023) and, as the tissue changes to fibrocartilage, become more isotropic (Fisher et al., 1985).

### 5.4 Lateral and medial capsule

The collagen orientations in the lateral and medial capsule are observed to be related to the direction of the collateral and accessory collateral ligaments. The collateral ligaments have a proximal‐distal orientation and are more cord‐like, but the accessory collateral ligament orientation lies in a transverse plane (Andrew, 1991; Slattery et al., 2014). Both Kuczynski (1968) and Allison (2005) describe this accessory ligament as a triangular fan‐shaped structure, but it is also capsular tissue and can be clearly seen from the inside of the capsule (Gad, 1967) (Figure 1). The accessory collateral ligaments are less substantial in structure than the collateral ligaments (Allison, 2005) and Slattery and colleagues were unable to definitively visualise these structures in dissections (Slattery et al., 2014).

In some early papers, there is the description of a triangular structure that penetrates the joint cavity, which was referred to as the meniscus (Fisher et al., 1985; Gad, 1967; Ralphs & Benjamin, 1994). However, later papers using high‐field‐strength magnetic resonance imaging (MRI) discerned that these structures were not fibrocartilaginous in nature and therefore decided to refer to these triangular structures as synovial folds (Lewis et al., 1996).

## 6 CAPSULAR ENTHESES

The enthesis is the site of attachment of a ligament, tendon, or capsule to the bone. This can be found as one of two types, either fibrous or fibrocartilaginous (Apostolakos et al., 2014; Benjamin et al., 2002). The capsular entheses have been shown to be important for force transfer from the capsule and the attached ligaments to the opposing phalanges. Both types of entheses are anisotropic as the fibers attach to the periosteum (fibrous) and bone (fibrocartilaginous) (Apostolakos et al., 2014; Benjamin et al., 2002). The main forms of collagen identified in these areas included types I, II, III, X (Apostolakos et al., 2014).

The proximal capsule enthesis is fibrous and associated with attachment via Sharpey's fibres to the periosteum (Milz et al., 2002). This fibrous enthesis develops in a proximal–distal orientation, with multi‐axial collagen penetrations into the bone insertion site (Aaron, 2012; Apostolakos et al., 2014; Benjamin et al., 2006; Whitebone et al., 2021).

Conversely, the distal capsule enthesis is fibrocartilaginous. A fibrocartilaginous enthesis can be identified by a thin layer of fibrocartilage containing type II collagen that connects the capsule, ligament, or tendon to bone (Adamczyk et al., 2008). The classic example of this is the calcaneal tendon enthesis (Milz et al., 2002). The palmar ligament is proximal–distal in orientation and highly anisotropic, transitioning from uncalcified fibrocartilage to bone (Benjamin et al., 1995, 2004, 2006; Benjamin & McGonagle, 2009). It is the main load transfer mechanism between the capsule, ligament, and the attachments.

## 7 IMPACT OF INJURIES AND SURGICAL INTERVENTION ON COLLAGEN ORIENTATION IN THE FINGER JOINTS

There are three important concepts that we must understand before we can assess dysfunction in the fingers. They are contraction, contracture, and compaction. Contraction is related to the normal physiological functions of the joint and is usually related to the muscles. Contracture is when the tendon or the tendon sheath stiffens, perhaps in response to aging, and becomes permanently tight, often resulting in flexion deformities and limited flexibility and movement of the digit. Compaction, on the other hand, is a result of a traction force exerted by the cells on the attaching collagen fibrils and results in the accumulation of fibrils around the cells.

A major cause of dysfunction in the human finger joints is the contraction of tendons and the joint capsule (Curtis, 1964). Most of the compaction occurs in the cross‐fibre direction (Ehrlich & Hunt, 2012). This is caused by tension, hydration, activation of the myofibroblasts and fibroblasts, and the resulting fibrotic actions which cause changes in Poisson's ratio, the ratio of lateral to longitudinal strain (Harris et al., 1981; Tomasek et al., 2002). The orientation of the collagen fibres determines the direction of the contracture. Collagen fibres and fibroblasts in tissue are oriented along the directions of stress (Tomasek et al., 2002). Fibroblasts are specialised cells which play a crucial role in wound healing and are released during the proliferative and remodelling phases (Cialdai et al., 2022). They are triggered in response to inflammatory cytokine release and aid in tissue healing and scar formation. In matrices which are under tension, the fibres form an hourglass shape perpendicular to the plane of stress (Tomasek et al., 2002).

Fibroblasts can differentiate into myofibroblasts during tissue repair (Bochaton‐Piallat et al., 2016; Castella et al., 2010). Myofibroblasts are able to repair injuries and do this by the generation of new fibres (Desmoulière et al., 2005; Gabbiani, 2003). As a result of new fibres being formed, there is compaction between the fibrils by pulling them together through the elimination of water and residual space, primarily across the fibre orientation (Adeeb et al., 2004; Ehrlich & Hunt, 2012).

Contracture is a common result of the body attempting to repair an injury, and prolonged myofibroblast results in the increased transcription of TGF‐β1, OB‐cadherin, and α‐SMA, similar to that observed in Dupuytren's contracture (Bochaton‐Piallat et al., 2016; Duscher et al., 2014; Phan, 2008; Tomasek et al., 2002; Verhoekx et al., 2013). Increased expression of these anabolic factors results in paracrine stimulation of the fibroblasts, resulting in further tension on the collagen structure. The increased tension leads to increased stiffness and sclerosis of the tissues as the repair takes place (Bochaton‐Piallat et al., 2016; Duscher et al., 2014; Gabbiani, 2003).

Stiffening and sclerosis of the tissue can typically present as a flexion deformity in the proximal–distal direction of the phalanges (Andrew, 1991; Crowe et al., 2020; Tonkin et al., 1985; Tuffaha & Lee, 2018). A flexion deformity of the finger occurs when the fingertip flexes due to tendon injury or bony avulsion, and the tip cannot be extended (Crum et al., 2022). The lengthwise changes in collagen fibre structure only take place in response to a muscle contraction; activation of the MMPs and collagenases causes cleavage, and the loss of fibre crimp due to excessive heat (such as that found in laser therapy) (Caley et al., 2015; Selecky et al., 1999; Vangsness Jr. et al., 1997).

A good example of the effect of cross‐directional contracture is demonstrated by the contraction of the A1, A3, and A5 flexor tendon pulleys. These are thickened areas of the flexor tendon sheath that play a role in flexion; tendon tracking and maintenance of apposition of the tendons and bones across joints, as well as a fulcrum for eliciting movement (Ashour et al., 2018; Beyermann et al., 2004; Blazar et al., 2016). As these tighten and contract in response to trauma or disease pathogenesis, they bear down on the flexor tendons, restricting movement perpendicular to the contraction and potentially even causing flexion (Saldana, 2001). The flexion can be surgically released via fasciectomy, therefore providing rapid resolution (Beyermann et al., 2004; Blazar et al., 2016; Tonkin et al., 1985; Tuffaha & Lee, 2018).

## 8 DISCUSSION

The structures of the human finger joint capsules and the surrounding tissues are complex and therefore determining the orientation of collagen fibres within the tissue is challenging. Due to the small size of the structures in question, there is a paucity of data relating to the collagen orientation. Additional difficulties in measuring the collagen fibre orientation include the fact that there are multiple layers of collagen fibres and the large range of the motion of the digits. The combination of these factors makes determining the fibre orientation technically very challenging.

The first question that we wanted to address in this review was: what are the main collagen orientations in the interphalangeal joints of the hand? There was limited evidence available despite exhaustive literature searches. The data that we did find indicated that the orientation was mainly proximal–distal, but there were some exceptions where the fibres were observed to be in the transverse orientation (Slattery et al., 2014; Watanabe et al., 1994). None of the studies we found indicated the angular distribution of the collagen fibres within the various tissues. The paucity of data may be related to the difficulty of dissecting the finger joints. It was evident from the literature that collagen fibres play an important role in strengthening the joint capsule and the pathological stiffness of the PIP joint (Bowers, 1981).

Our second question about the relationships between collagen fibre orientation and joint function showed that they are closely related, with the direction often following the direction of joint/tissue loading and related to the function of the joint (Ralphs & Benjamin, 1994). The main example that we found evidence for was the palmar ligament which had fibres running in a longitudinal direction. For many of the other structures within the joint capsule described in this paper, there is very little evidence in the literature about the collagen fibre orientation and the dominant direction.

The final question that we wished to address was how common surgical interventions affect collagen orientation in the IP joint capsules and what the functional outcomes are after surgery. It has been shown that the direction of incisions in the skin and the finger joint capsule affects the healing of the tissue at the incision site and joint movement. When an incision follows the collagen orientation, the wound is less likely to gape and will heal better with less formation of scar tissue resulting from cutting the collagen fibres and causing compaction. It should be noted, though, that in some cases incisions across the fibres cannot be avoided (Haase & Chung, 2014; Watson et al., 1979). Surgeons have shown that incisions in the sagittal plane through the proximal‐distal dorsal extensors, which follow the collagen orientation, have shown better healing and surgical outcomes (Afifi et al., 2010). However, a large proportion of surgeons favor palmar and lateral approaches, which avoid the risk of compromising the extensor apparatus (Duncan et al., 2018; Trumble & Heaton, 2017). These approaches release palmar adhesions, a band of fibrous scar tissue that may join two usually separate surfaces, which may cause fixed flexion deformities. A fixed flexion deformity can occur when the digit remains permanently bent and affects the function of the digit (Shih & Bayat, 2010). As the main issue is a contracture of the collagen fibres, there are several ways to treat such a deformity, including splinting, exercises, and in extreme cases, surgical incisions (Shih & Bayat, 2010).

The limited evidence from the literature highlights the incomplete nature of the descriptions of the joint capsule, which may in part be due to its complex three‐dimensional structure and its small size. There are some excellent early studies which include detailed descriptions of the joint capsule along with some useful images (Fisher et al., 1985; Lee et al., 2014; Slattery, 1990) but our understanding has advanced little since these studies, although tools for dissection and visualization have improved considerably. Due to the irregular internal shape of the capsule, it is difficult to determine the optimal sampling position for measuring capsule thickness and collagen fiber orientation. We know that incisions across the fibers are less likely to heal well than incisions aligned with the fiber orientation (Carmichael, 2014; Langer, 1978; Ní Annaidh et al., 2012). Therefore, improving our understanding of collagen orientation and the effects that it has on the movement of the finger joints and wound healing warrants further investigation.

## AUTHOR CONTRIBUTIONS

R.G.C., F.R.S., R.M.A. & F.G. contributed to the drafting of the manuscript, critical revision, and approval of the manuscript.

## Data Availability

Data sharing is not applicable to this article as no new data were created or analyzed in this study.
